# Phenological variation of flower longevity and duration of sex phases in a protandrous alpine plant: potential causes and fitness significance

**DOI:** 10.1186/s12870-020-02356-w

**Published:** 2020-04-03

**Authors:** Zhigang Zhao, Meng Hou, Yijie Wang, Guozhen Du

**Affiliations:** State Key Laboratory of Grassland and Agro-Ecosystems, School of Life Sciences, Lanzhou University, Lanzhou, 730000 China

**Keywords:** Flower longevity, Phenotypic sex, Sex allocation, Phenology, Protandrous, *Aconitum gymnandrum*

## Abstract

**Background:**

Flower longevity plays an important role in pollen dispersal and reproductive success in plants. In dichogamous plants, the duration of anthesis as well as the time allocated to male and female functions can vary in response to intrinsic factors (e.g., flowering time and resource allocation) and pollination context along a growth season. However, the fitness consequences of phenological dynamics have rarely been examined. This study aims to unravel the potential causes driving variation in flower longevity, duration of sex phases, and phenotypic sex during a flowering season of strongly protandrous *Aconitum gymnandrum*, and particularly reproductive consequences of the phenological pattern.

**Results:**

Population floral sex ratio shifted from completely male at the beginning to completely female at the end of the season, as is common in other protandrous plants. Phenological dynamics of the floral sex ratio and the duration of sex phases caused a shift from femaleness to maleness in the mean phenotypic sex over the whole season. Floral longevity was negatively correlated with flower size and positively affected by temperature. Early flowers within inflorescences rather than early-flowering individuals emphasized the duration of female over male phase. Owing to the dominance of male-phase flowers, early flowering for individual flowers and plants, or female-biased sex resulted in higher pollen deposition per flower and seed set. At the flower level, flower longevity positively affected female reproductive success, while the effect of flower size was negative. By contrast, plant-level female reproductive success was negatively affected by flower longevity but positively correlated to flower size.

**Conclusions:**

The major result of this study lies in elucidating the relationship between variation in phenological sex expression and floral longevity and their fitness consequences of protandrous *A. gymnandrum*. The contrasting results on female fitness for individual flowers and plants contribute to our current understanding of the adaptive significance of floral longevity.

## Background

Floral longevity, which is the length of time flowers remain open and functional, plays an important role in pollen removal and deposition due to its effect on floral display size, number of pollinator visits, and eventual reproductive success in flowering plants [1, 2]. Longer flower lifespan is often associated with pollinator attraction in the face of infrequent and unpredictable pollination [3–8], such as in alpine environments. Flower longevity is also sensitive to other environmental factors such as water availability and air temperature [1, 9–13]. As flower development and maintenance demand carbon, nutrients and water resources [1, 14, 15], extended longevity may bring high energetic and transpirational costs to the plant in order to maintain flower function [14]. Therefore, flower longevity should be optimized by selection to maximize fitness at minimum costs to the maternal and paternal plants [2, 16]. Flower longevity often varies among species, among populations, among individuals within a population, and even among flowers within individuals [1, 17, 18]. To understand the potential adaptive significance of flower longevity, it is necessary to disentangle potential causes associated with seasonal flower lifespan variation within a species.

As a part of the flower display trait, plastic floral longevity in response to variable environmental conditions can favour fitness accrual rates and would therefore be adaptive [2, 11, 19]. In dichogamous species with separate male and female phases, the duration of the sex phases may be more plastic and can also be optimized by selection to balance pollen transfer and floral maintenance costs. Since the maintenance of flower functioning consumes resources, flower size or flower number could intrinsically influence any adaptive alterations in flower longevity or the duration of male and female phases when available resources are limited. In addition, dichogamy can strongly affect the mating environment of flowers and therefore the reproductive success of plants in a population [20, 21]. Predictions based on resource allocation to sexual functions [20–22] can also be extended to optimal time allocation to male and female phases in dichogamous flowers. Protandrous plants produce male-phase flowers prior to female phase, thus causing a shift of population sex ratio and dynamic phenotypic sex of individuals from pure or primarily male to pure or primarily female during the flowering season. When early flowers in protandrous plants shed pollen, relatively fewer stigmas are available; consequently, competition for ovules reduces the probability of pollen transfer and siring success. Thus, female reproductive success can be maximized by higher pollen availability due to a male-biased population sex ratio at the beginning of a flowering season. In contrast, at the end of the season, reduced pollen availability in male phase flowers can decrease pollen deposition and may reduce female reproductive success irrespective of resource availability. There should be more time allocated to the female phase for early flowers within plants or early flowering plants. Given the varied flower longevity and phenotypic sex, few studies have evaluated temporal allocation to male and female phases of flowers [23].

Considering the whole growing season, dichogamous plants that flower early or late may encounter different resource conditions and thus would be affected by intrinsic factors (e.g., resource allocation) and pollination context [23]. To date, studies have addressed the effects of mating opportunities and climatic factors underlying phenological patterns in order to understand the role of plasticity in floral sex phase duration and sex expression in dichogamous plants [23–25], but little attention has been paid to the seasonal dynamics of reproductive performance in order to further discriminate fitness consequences of phenology-associated phenotypes. In contrast to the plastic response of flowers to environmental factors, efforts to clarify the potential adaptation and evolution of flower longevity as well as the duration of male and female phases and sexual expression of individuals should focus on the association of among-plant variation with reproductive success [26].

The overarching goal of this study was to examine phenological patterns in the duration of the floral sex phase and its impact on reproductive performance. To address this question, we examined seasonal changes and possible causes of floral sex phase duration, longevity, and phenotypic sex in strongly protandrous *Aconitum gymnandrum* on the eastern Tibetan Plateau in China, and subsequently elucidated their potential effects on reproductive success. Specifically, we addressed the following questions:

1. What is the pattern of temporal variation in flower longevity, duration of male and female phases, sex ratio, and phenotypic sex in a population of *A. gymnandrum*?
2. What is the relative role of intrinsic vs. extrinsic (climatic) factors affecting floral longevity as well as the duration of male and female phases during the flowering season?
3. How do floral longevity, flowering time, and phenotypic sex affect female reproductive success? We expect increased female fitness for extended flower longevity (especially the female duration) and for early flowering of individual flowers and plants in the alpine environment of Tibetan Plateau.

## Results

### Temporal variations in flower longevity, duration of male and female phases, sex ratio, and phenotypic sex

**Fig. 1:**
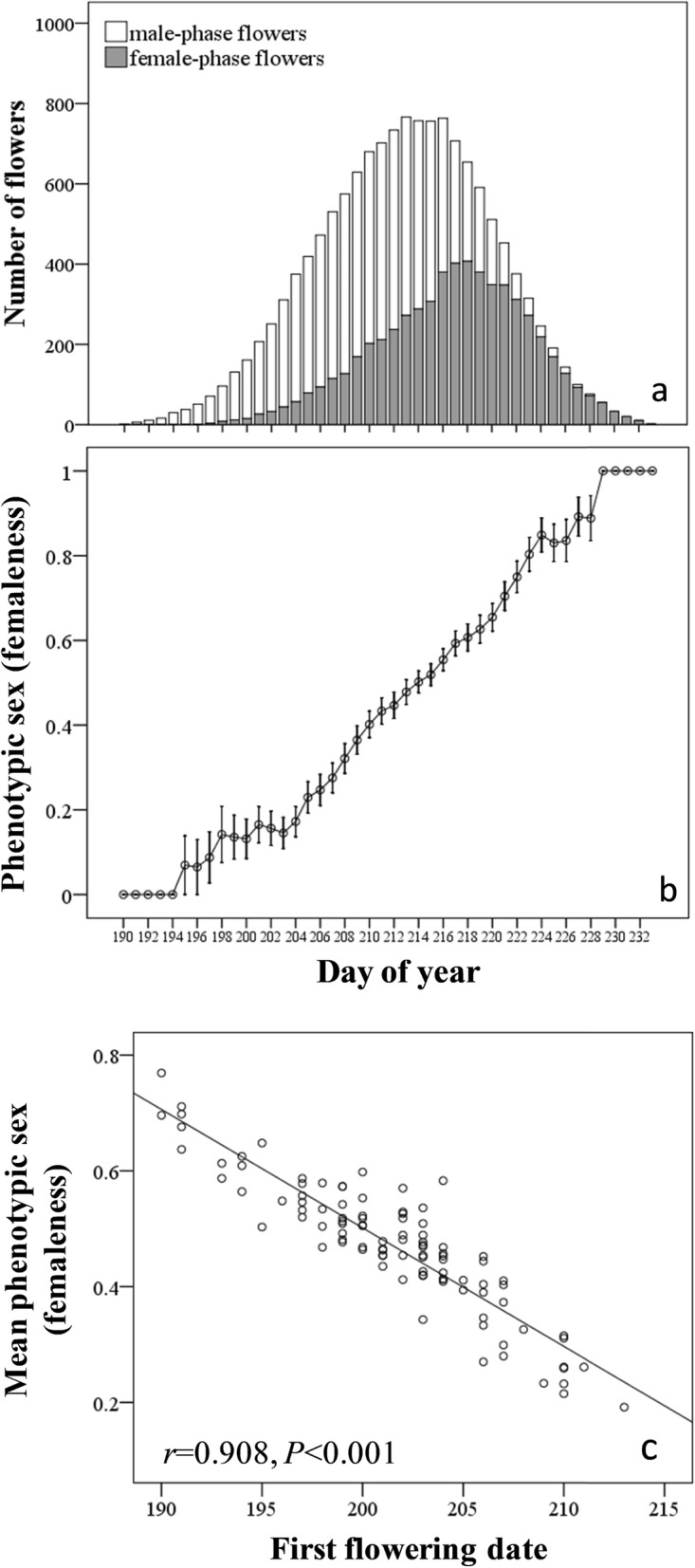
Flowering dynamics of *Aconitum gymnandrum* populations. **a** The number of male- and female-phase flowers along the season; **b** changes in the daily average phenotypic sex for the population (grey line, mean ± standard error); **c** the relationship between mean phenotypic sex and first flowering date of *A. gymnandrum* individuals

*A. gymnandrum* flowered from early July to late August and peaked from late July to early August. The flowering duration between individuals largely overlapped (Figure S2). During the flowering season, flower longevity ranged from 10 to 19 days with an average of 13.85 days (Figure S3). Flowers remained in the male phase (8.4 ± 0.05 days) longer than in the female phase (5.4 ± 0.05 days) (*F*_1,1193_ = 2000.1, *P* < 0.0001). Population floral sex ratio during the season, i.e., the number of male-phase flowers versus female-phase flowers, shifted from completely male at the beginning to nearly 1:1 at peak and completely to female at the end (Fig. 1a). Since the male duration was longer than the female duration, female phase flowers were present less frequently than male phase flowers (Fig. 1a). The daily mean phenotypic sex of the population changed from maleness to femaleness over the course of the flowering period (Fig. 1b). A plant’s mean phenotypic sex over the whole season was negatively related to its first flowering date (Fig. 1c).

### Factors influencing floral longevity and duration of male and female phases

**Fig. 2:**
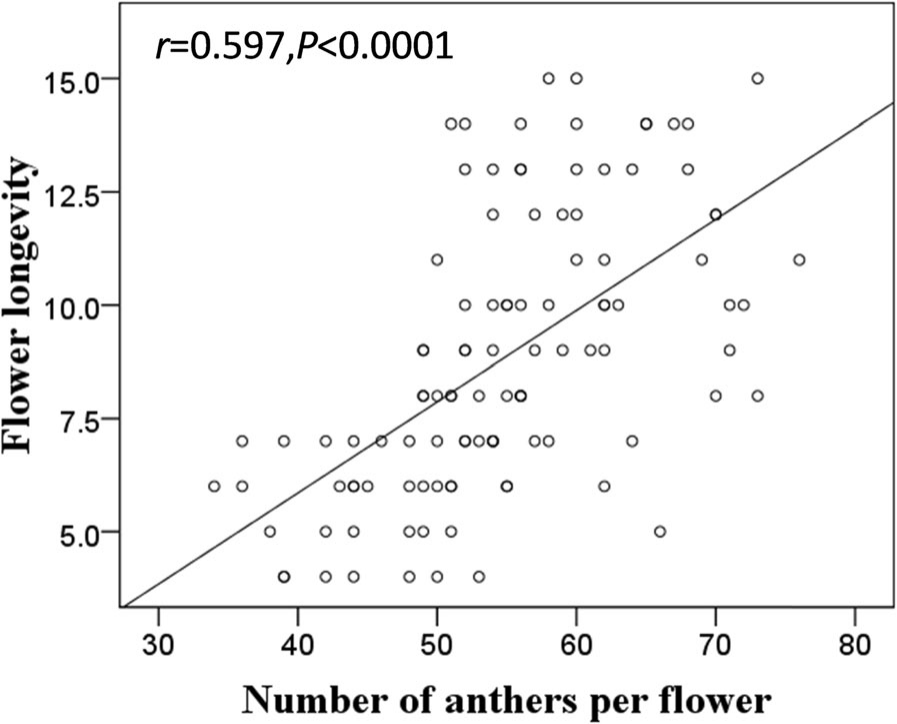
The relationship of single flower longevity and anther number per flower (*N* = 107)

Flower longevity was positively correlated with the number of anthers (Fig. 2) and mean temperature (Table 1), and negatively correlated with flower size (Table 1). The first flowering date of individuals (FFD) and the relative flowering date of individual flowers within plants (RFD) as well as flower number did not affect flower longevity (Table 1). Most of the predictors were correlated with each other (Figure S4). A negative relationship between the FFD and RFD with flower size showed that early flowers were larger.

**Table 1:** Potential predictors affecting flower longevity and the duration of male and female phases through a generalized linear mixed model analysis

Predictor	Flower longevity			Male duration			Female duration		
	Estimate	SE	*P*	Estimate	SE	*P*	Estimate	SE	*P*
FFD	−0.227	0.134	0.090	−0.100	0.078	0.202	−0.110	0.080	0.227
RFD	−0.005	0.076	0.950	0.157	0.048	**0.001**	−0.125	0.058	**0.030**
Flower size (mm)	−0.178	0.066	**0.007**	−0.173	0.051	**< 0.001**	−0.035	0.052	0.503
Flower number	0.111	0.116	0.341	0.009	0.073	0.904	0.102	0.073	0.165
Mean temperature (°C)	0.542	0.108	**< 0.001**	0.154	0.078	**0.048**	0.283	0.085	**< 0.001**
Mean precipitation (mm)	0.168	0.100	0.092	0.258	0.070	**< 0.001**	−0.104	0.058	0.072

*FFD* first flowering date of individuals, *RFD* opening day of flowers relative to a plant's day of flowering onset; flower size is galea height. Mean temperature and mean precipitation are values experienced by that flower over the days it was open. The parameters were estimated by Generalized linear mixed models.

The RFD was positively correlated with the duration of male phase, but negatively correlated with the duration of female phase; the FFD was not related to duration of the male or female phases (Table 1). Mean temperature positively affected duration of both sex phases, but mean precipitation and flower size only influenced duration of the male phase (Table 1).

Artificial pollen removal and deposition did not affect duration of the male phase (*F*_1,78_ = 0.028, *P* = 0.869), duration of the female phase (*F*_1,78_ = 1.904, *P* = 0.172) (Figure S5), or total flower longevity (*F*_1,78_ = 2.375, *P* = 0.127).

### Factors associated with flower- and plant-level female reproductive success

At the flower level, pollen deposition was negatively correlated with the FFD, RFD, and duration of the female phase, and positively correlated with flower number (Table 2). Seed set was negatively correlated with the FFD, RFD, and flower size, and positively correlated with floral longevity (Table 2).

**Table 2:** Effects of flower and plant traits on the components of reproductive success (pollen deposition per flower and seed set per fruit) of individual flowers of *Aconitum gymnandrum*

Predictor	Pollen deposition			Seed set		
	Estimate	SE	*P*	Estimate	SE	*P*
FFD	−0.211	0.057	**< 0.001**	−0.596	0.142	**< 0.001**
RFD	−0.335	0.069	**< 0.001**	−1.041	0.163	**< 0.001**
Female phase (day)	−0.031	0.005	**< 0.001**	**–**	**–**	**–**
Flower longevity (day)	**–**	**–**	**–**	0.268	0.052	**< 0.001**
Flower size (mm)	0.037	0.005	0.070	−0.167	0.043	**< 0.001**
Flower number	0.102	0.056	**0.048**	−0.008	0.141	0.957

*FFD* first flowering date of individuals, *RFD* opening day of flowers relative to a plant’s day of flowering onset; flower size is galea height. The parameters were estimated by Generalized linear mixed models

**Fig. 3:**
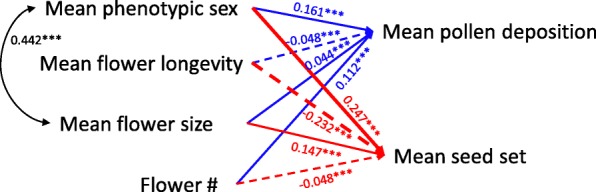
Effects of plant traits (mean measurements for a plant) on the components of reproductive success (mean pollen deposition per flower and mean seed set per fruit) of *Aconitum gymnandrum.* The values on single arrows represent the partial regression coefficients estimated by Generalized linear mixed models. The value on double arrow represent the correlation coefficient between two traits. See statistical analysis section for details. Solid lines indicate positive relationships and dashed lines indicate negative relationships; nonsignificant correlation lines were not shown.*** indicates *P* < 0.001

At the plant level, mean pollen deposition per flower was positively correlated with female-biased sex, flower size, and flower number, and negatively correlated with flower longevity (Fig. 3). Mean seed set was positively correlated with female-biased sex and flower size, and negatively correlated with flower longevity and flower number (Fig. 3). Female-biased sex was positively correlated with mean flower size (*r* = 0.442, *P* < 0.001, Fig. 3). There was no relationship between mean pollen deposition on a flower and mean seed set per fruit in *A. gymnandrum* individuals (*r* = 0.068, *P* = 0.504, *N* = 97).

## Discussion

### Phenological variations in floral longevity, duration of male and female phases, phenotypic sex, and possible factors

Floral sex ratio, but not flower longevity, changed during the flowering season in the protandrous *A. gymnandrum* population. In combination with dichogamy and the dynamics of population sex ratio, the daily phenotypic sex of the population shifted from male-biased at the beginning of the season to female-biased at the end of the season. Correspondingly, a plant’s mean phenotypic sex changed from femaleness to maleness over the whole season. This seasonal pattern would cause within- and among-plant variation in floral allocation to duration of sex phases, which is consistent with the model based on resource allocation to sexual functions proposed by Brunet and Charlesworth [21]. According to our expansion of resource allocation predicted by the model [21, 23] to time allocation, early flowers within an inflorescence emphasized the female vs. the male phase. As the opposite occurred to late flowers within an inflorescence, no net change in floral longevity was observed within the plants. Contrary to our expectations, the duration of the female and male phases was not related to the FFD. The lack of influence of the FFD suggests that within-inflorescence changes of duration of the male vs. female phases might be enough to produce the adjustment of individuals to the pollination context, i.e., late flowering plants start producing already flowers with the male duration that fits the pollination context at that late flowering dates, particularly because there was wide overlap between individuals in their flowering duration.

Seasonal variation in floral sex allocation should be similar in terms of a plant’s commitment of resources or time. Consistent with the hypothesis, Ishii and Harder [23] found higher female investment in both resources and time for early-flowering plants and early flowers within individuals of the protandrous *Delphinium glaucum*, which was associated with a shift in the floral sex ratio of the population from male- to female-biased. In another study, Aizen [26] did not find any changes in relative temporal allocation to the duration of male and female sex phases within flowers or in resource allocation into pollen and ovule production throughout the flowering period in the protandrous *Alstroemeria aurea*.

As expected, the negative effect of flower size on flower longevity revealed that *A. gymnandrum* individuals cannot sustain both larger and long-lived flowers, which is in line with other studies [4, 27, 28]. Larger displays expend more resources for maintenance and pollinator attraction [2, 29], thus the negative relationship occurs when increased longevity of flowers would increase resource consumption [27]. Floral maintenance costs of *A. gymnandrum* might be related to nectar production because the nectar reward is an important trait for pollinator visitation and is produced throughout the life of an individual flower [30]. Models of floral longevity have assumed allocation of limited resources to either floral maintenance or construction of new flowers [2]. A trade-off likely exists between flower longevity and flower number [19, 31], but it may occur between flower longevity and flower size for *A. gymnandrum*.

A flexible flower longevity is theoretically favoured to ensure reproductive success [1]. A negative relationship between pollination and flower longevity has frequently been reported in other species [16, 31–33], but we found no evidence of an effect of artificial pollination on flower longevity in *A. gymnandrum*. This may be related to the slower anther dehiscence of *A. gymnandrum* flowers. Only a few of the dozens of anthers dehisce every day (Zhao Z.G. pers. obs.), which results in a positive correlation of single flower longevity with anther number independent of flowering time. Aximoff and Freitas [34] also showed that the longevity of anthesis in *Salvia sellowiana* was related to the gradual dispensing of pollen, in which flower longevity did not vary between hand-pollinated and open-pollinated flowers, although they determined that excluding pollinators significantly extended longevity. The staggered dehiscence of anthers may limit pollen removal per visit by individual pollinators according to pollen-presentation theory [35]. It is favoured when pollinators are abundant or the frequency of visits was highly variable [36]. The association of anther dehiscence mode and the possibility of pollen dispersal shows that the duration of the male phase can be subject to selection through male reproductive success. Floral female phase duration of *A. gymnandrum* also did not respond to pollen deposition. Persistent female function might help to maintain floral attractiveness for pollinators at the plant level, especially when seed production is pollen-limited. This is possible because the late stage of the floral female phase of *A. gymnandrum* still has high nectar production [37]. A similar pattern of remaining open and continuing to secrete nectar after pollination has also been found in the protandrous *Alstroemeria aurea* [38].

During the growing season, asynchronously flowering individuals would face fluctuating extrinsic environmental conditions. Considering abiotic factors, flower longevity of *A. gymnandrum* was more affected by temperature than precipitation. However, flower longevity increased with temperature via its effect on the duration of male and female phases, which is contrary to other studies that reported a negative effect of temperature [7, 9, 11, 25]. Longer flower duration, and consequently more opened flowers on a plant may possibly benefit from increased pollinator visitation due to high temperature. Our previous study found that *A. gymnandrum* populations in the study site experienced pollen limitation [30], but longer flower duration may have a high maintenance cost, e.g., extended nectar production for pollinator attraction. A possible solution is that a reduction in flower size could be accompanied with an extended flower longevity, which could alleviate maintenance costs to some extent.

### Fitness consequences of flower longevity, flowering phenology, and phenotypic sex

Flower longevity, phenology, and phenotypic sex had independent effects on reproductive performance. We found that flower longevity had opposite effects on female fitness at the flower and plant levels. The association of flower longevity with fitness could reflect its role in response to specific mating opportunities and should therefore be shaped by natural selection [2, 7]. For example, enhanced female fitness was associated with extended flower longevity in *Rhododendron calendulaceum* [39] and *Narcissus serotinus* [40] under pollen limitation, or poor pollinator service. Using a manipulation experiment, Rathcke [16] also demonstrated that prolonged floral longevity increased the female reproductive success of *Kalmia latifolia*. Similarly, at the flower level, flower longevity of *A. gymnandrum* had a positive effect on seed set while it was negative for flower size, showing that flower duration associated with mating opportunity in a population is vital for the pollination success of individual flowers of *A. gymnandrum*. Contrary to our expectation in the alpine environment, floral female phase duration was negatively associated with pollen deposition. This seems to contradict the results of the artificial pollination treatment, in which artificial pollen removal and addition did not affect the duration of the male and female phases. There was an important discrepancy between artificial and natural pollination for the plant, i.e., our artificial pollination did not extract nectar produced by the flowers. Therefore, artificial pollination did not result in any energetic cost; in contrast, natural pollination incurred the cost of renewing nectar removed during insect visitation. In orchid species, for example, flowers with rewards are more physiologically expensive than deceptive ones, and thus close sooner after pollen removal in order to reduce flower maintenance costs [41].

At the plant level, by contrast, larger flowers enhanced the mean pollen load per flower and the mean seed set of *A. gymnandrum* individuals, while the mean flower longevity had a negative effect on the female fitness component. In this species, larger flowers have more nectar and could provide an honest signal of rewards for pollinators [37]. The association of flower size with pollinator attraction and reproductive success of plants has frequently confirmed pollinator-mediated phenotypic selection towards larger flowers [42–45]. Out results show that larger and shorter-lived flowers in *A. gymnandrum* individuals are favoured by selection, which is the opposite of the flower-level pattern.

As expected, flowering time and phenotypic sex played relatively more important roles in determining female reproductive success of *A. gymnandrum* flowers and individuals. Early anthesis for individuals and flowers within inflorescences had more pollen deposited per flower on average and thus increased seed set. As male phase flowers were dominant in the beginning of the flowering season, they provided high pollen availability to maximize female fitness. Similarly, van der Meer and Jacquemyn [46] found that early flowers and early-flowering plants produced more seeds per fruit in *Saxifraga granulata.* Brookes and Jesson [22] also found that the early-flowering and male-biased sex ratio was positively correlated with female fitness in protandrous *Stylidium armeria*. In this study, the high seed set for early-flowering plants was in line with many studies that have shown significant selection for early flowering [47–51]. Interestingly, we found that the phenotypic sex of *A. gymnandrum* individuals affected reproductive success independent of flower display traits. According to the observed shift in the population sex ratio, plants exhibiting female-biased sex, i.e., those that flowered relatively early in the population, received more pollen on average and set more seeds. Similarly, a higher density of male-phase flowers and a lower density of female-phase flowers increased the seed production of the protandrous *Lobelia cardinalis* population [52].

Considering pollination context, temporal variability in the abundance and composition of pollinators has been suggested to obscure or even counteract potential deterministic effects of changes in pollen availability associated with the temporal shifts in sex-ratios of protandrous plants [53, 54]. Female function can be completed after a single pollinator visit compared to male reproductive success, which can continue to increase as more pollen grains are removed from a flower or the whole plant [55]. An association of increased pollen dissemination (i.e. male reproductive success) with longer flower longevity has been reported in *Aquilegia buergeriana* var. *oxysepala* [56] and *Salvia sellowiana* [34]. Although the pattern of flowering dynamics and phenotypic sex in *A. gymnandrum* is similar to other protandrous species [21, 23, 26], our findings on the determinants of female reproductive success strongly show that the phenological patterns of flower longevity and phenotypic sex should be favored by selection. By contrast, as population sex ratio becomes more female towards the end of the flowering season, late flowering and/or male-biased sex should maximize male reproductive success of protandrous plants due to the high ovule availability. Therefore, more studies on the impact of flower longevity and flowering phenology on male fitness are needed to better understand sex specialization of protandrous species.

## Conclusion

The major finding of this study was that the phenological variation of flower longevity, duration of sex phases and plant phenotypic sex strongly affected female reproductive success of flowers and plants of protandrous *A. gymnandrum*. Within-population changes of floral sex ratio, which was the same as in other protandrous plants [23, 26], caused a shift of the plant’s mean phenotypic sex from femaleness to maleness over course of the whole season. Early flowers within inflorescences rather than early-flowering individuals emphasized the extended duration of the female over the male phase. As a result, early flowers and early-flowering individuals with female-biased sex, had higher pollen deposition per flower and seed set. The variation of flower longevity was associated with flower size and temperature rather than flowering phenology. Extended floral longevity but smaller flower size increased flower-level pollination success. However, at the plant level, individuals of *A. gymnandrum* with shorter-lived and larger flowers had higher female reproductive success. Our findings highlight the phenological pattern and fitness consequences of phenotypic sex plasticity in protandrous species. In particular, we found contrasting results for individual flowers and plants that shows an ability to adjust on the plant level for maximizing an individual’s fitness and contributes to our understanding of the adaptive significance of floral longevity.

## Methods

### System and study sites

*Aconitum gymnandrum* Maxim. (Ranunculaceae) is an annual herb widely distributed in alpine meadows (1600–3800 m) on the Tibetan Plateau, China. Plants commonly bloom from June through August. Individual plants usually produce one to several erect racemes consisting of 2–30 blue-purple zygomorphic flowers, which typically open sequentially from bottom to top (generally 4–7 flowers open at once), and are pollinated by bumblebees (at the study site mainly by *Bombus consobrinus* and *B. sushkini*) [30]. Each flower has 6–14 separate carpels (each with 8–14 ovules) surrounded by 30–90 stamens [57]. The galea (or hood), formed from one of the petaloid sepals, contains two stalked petals with nectaries inside, and two other petals extend and cover the stamens and carpel. *A. gymnandrum* is self-compatible and strongly protandrous like other species in the genus. The anthers dehisce over 4–5 days and stigmas become receptive 1–2 days after the end of anther dehiscence [58]. Fruit maturation requires 20–30 days.

We studied a population of *A. gymnandrum* located in the Alpine Meadows and Wetland Ecosystems Research Station of Lanzhou University (E102°53′, N34°55′, 2950 m a.s.l) on the eastern Tibetan Plateau, Gansu Province, China. *A. gymnandrum* is a native plant species and not endangered, collection of samples for scientific purposes was permitted by local legislation. Research permission was obtained for this project from the State Key Laboratory of Grassland and Agro-Ecosystems, School of Life Sciences, Lanzhou University. Dr. Zhigang Zhao undertook the formal identification of the samples. A voucher specimen has been deposited at the State Key Laboratory of Grassland and Agro-Ecosystems, Lanzhou University (voucher No. ps − 20,060,618–001).

### Flowering phenology and floral longevity

From July to August 2013, we characterized seasonal variation in flower longevity, flower size, sex ratio (female vs. male flowers), and plant phenotypic sex, and quantified pollinator visitation. Temperature and precipitation data were obtained from Hezuo weather station, less than 1 km away from our field station. The study population consisted of 586 individuals. Prior to anthesis we randomly tagged 100 plants and recorded the flowering day of all flowers of main inflorescences of individuals. A total of 1085 flowers were marked, and flower longevity was estimated as the duration of combined male and female phases (Figure S1). Anther dehiscence usually occurred soon after flower opening in this species. The male phase began as one anther dehisced and ended when all anthers dehisced and withered. At the end of the male phase, the stigma generally becomes receptive, and we regarded this as the start of the female phase. The end of the female phase was recorded when two stigma branches of each carpel curved down and the stigma surface became brown, accompanied by at least one sepal wilting. On each plant, we sampled two flowers from each of the bottom, middle, and top of the main inflorescence to measure flower size. The size of these six flowers was estimated by measuring the galea height (vertical distance from the base of the flower to the top of galea) using digital calipers (Mahr Federal 16 ER Digital Calliper, Germany) when they individually entered the middle of the male phase (i.e., half of anthers dehisced). In order to estimate the association of the anther number with single flower lifespan, 55 of the 100 plants were randomly selected, and the anther number of the two bottom flowers was counted.

We used flowering time and floral longevity data to calculate the daily population sex ratio and the phenotypic sex, and to examine possible causes associated with floral longevity and duration of the male and female phases.

### Pollen deposition and seed set

The six flowers sampled for estimating longevity on each plant were also used to determine components of female reproductive success. The first flower at each of three positions was used to estimate natural seed production (female reproductive success) when fruits matured (*N* = 296 fruits). The other flower from each position was used for recording stigmatic pollen loads. At the end of the female phase, the stigmas were cut off and mounted on slides and taken back to the lab. *Aconitum* pollen morphology was obviously different from other co-flowering species and easy to distinguish from interspecific pollen. The deposited pollen was counted directly under a microscope (Olympus BX53) on a total of 298 flowers; interspecific pollen grain loads (mainly *Pedicularis kansuensis* and *Taraxacum mongolicum*) were lower than 5%.

We used pollen deposition per flower and seed set data to test the associations of floral longevity, phenotypic sex, and flower phenology on female reproductive success.

### Effects of pollen removal and pollen receipt on the duration of male and female phases

We performed artificial pollinations to test whether high pollination levels can affect the duration of the male and female phases. Twenty plants of a similar size were marked near the studied population. For each plant, four flowers at the bottom of the main inflorescences (some individuals had 1–3 lateral inflorescences) were chosen: the first and third flowers received the artificial pollination treatment, while the second and fourth served as controls. As the flowers in the artificial pollination treatment came into the male phase, we gently brushed the dehisced anthers using a fine hairbrush twice every day (in the morning and afternoon). When they entered the female phase, we used dehisced anthers from other plants to touch receptive stigmas twice every day (in the morning and afternoon) until the female phase ended. Flowers were bagged prior to treatment to prevent pollinator access.

### Statistical analysis

All statistical analyses were conducted in R version 3.2.3 [59].

### Temporal variation in sex ratio and phenotypic sex

The daily population sex ratio was estimated as the number of male divided by the number of female flowers. As the daily floral sex ratio does not consider the mating opportunities for female and male success (which depend on the population floral sex ratio [60, 61]), we estimated the standardized phenotypic sex that incorporates the relative availability of pollen and ovules in the whole population as [60]:

$$ {G}_{i,t}=\frac{f_{i,t}}{f_{it}+{m}_{i,t}{E}_t}, $$

Where *G*_*i*, *t*_ is the phenotypic sex of plant *i* on day *t*, *f*_*i*, *t*_ and *m*_*i*, *t*_ are its numbers of female- and male-phase flowers, respectively, of plant *i* on day *t*, and ${E}_t=\frac{\sum \limits_i{f}_{i,t}}{\sum \limits_i{m}_{i,t}}$. *E*_*t*_ shows the relative opportunity of male-phase flowers to sire seeds for a plant when considering population context, as an equivalence factor. Mean phenotypic sex for each plant over the whole season can be estimated as:

$$ {\overline{G}}_i=\frac{\sum_t{G}_{i,t}{n}_{i,t}}{\sum_t{n}_{i,t}}, $$

where *n*_*i,t*_ is the number of flowers opened by plant *i* on day *t*.

### Potential causes affecting floral longevity and the duration of sex phases

We used Generalized linear mixed model to estimate the magnitude of potential factors affecting floral longevity and duration of the male and female phases. In the models, predictors included the first flowering date of individuals (FFD), the day of flower opening relative to the plant’s day of flowering onset (RFD), flower size (galea height) and flower number per plant, the mean air temperature, and precipitation experienced by that flower over the days it was open. Since our dataset had a nested structure and flower-level data because response variables were not fully independent, we considered variation within plants by including plant ID within the population as a random effect in the models. The effect of hand pollination on duration of male and female phases was examined by ANOVA.

### Effects of flower longevity, phenology and phenotypic sex on female reproductive success

We conducted generalized linear mixed models at the flower and individual levels to determine the relative effects of flower longevity and other display traits, phenology, and phenotypic sex on the pollen number deposited per flower and seed set. All traits were standardized for a comparison between regression coefficients in the models. In the flower level analysis, when pollen deposition per flower was the response variable, we included the standardized duration of the female phase, flower size, RFD, FFD, and flower number as predictors, and plant ID as a random effect. When seed set was used as the response variable, we included the standardized flower longevity, flower size, RFD, FFD, and flower number as predictors, and plant ID as a random effect. In both models, Poisson error distribution was assigned to response variables. The analyses included 295 flowers on 99 plants. In the plant level analysis, we used mean pollen deposition per flower and mean seed set per fruit as response variables, both with Poisson error distribution. In each model, we used the standardized mean phenotypic sex of individuals, mean flower longevity, mean flower size, and flower number as predictors, and plant ID as a random effect. As the flowers used to score pollen deposition on stigmas were not the same as those used for seed production, we further estimated the relationship between mean pollen deposition per flower and mean seed set of individuals by a regression analysis.

## Supplementary information

**Additional file 1 **: **Figure S1.** The male and female phase of protandrous flowers of *Aconitum gymnandrum*.

**Additional file 2 **: **Figure S2.** Number of plants by the flowering duration throughout the season.

**Additional file 3 **: **Figure S3.** The distribution frequency of flower longevity.

**Additional file 4 **: **Figure S4.** The correlation among predictor variables affecting flower longevity and their distribution. FFD = first flowering date of individuals, RFD = opening day of flowers relative to the plant’s day of flowering onset; flower size is galea height. Mean temperature and mean precipitation are values experienced by that flower over the days it was open. *P < 0.05, **P < 0.01, ***P < 0.001.

**Additional file 5 **: **Figure S5.** The effects of pollen removal and pollen deposition on sexual phase durations. (White box is the control, gray box is the pollination treatment).

## Data Availability

The datasets used and/or analysed during the current study are available from the corresponding author on reasonable request.

## Abbreviations

FFD: First flowering date of individuals
RFD: Relative flowering date of flowers
