# Supplementary material for: Phenological variation of flower longevity and duration of sex phases in a protandrous alpine plant: potential causes and fitness significance
Source: BMC Plant Biol. 2020 Apr 3;20:137. doi: 10.1186/s12870-020-02356-w (PMC7118941; doi:10.1186/s12870-020-02356-w)
Supplement: Supplementary file 4 — Additional file 4 : Figure S4. The correlation among predictor variables affecting flower longevity and their distribution. FFD = first flowering date of individuals, RFD = opening day of flowers relative to the plant’s day of flowering onset; flower size is galea height. Mean temperature and mean precipitation are values experienced by that flower over the days it was open. *P < 0.05, **P < 0.01, ***P < 0.001. [file 12870_2020_2356_MOESM4_ESM.docx]

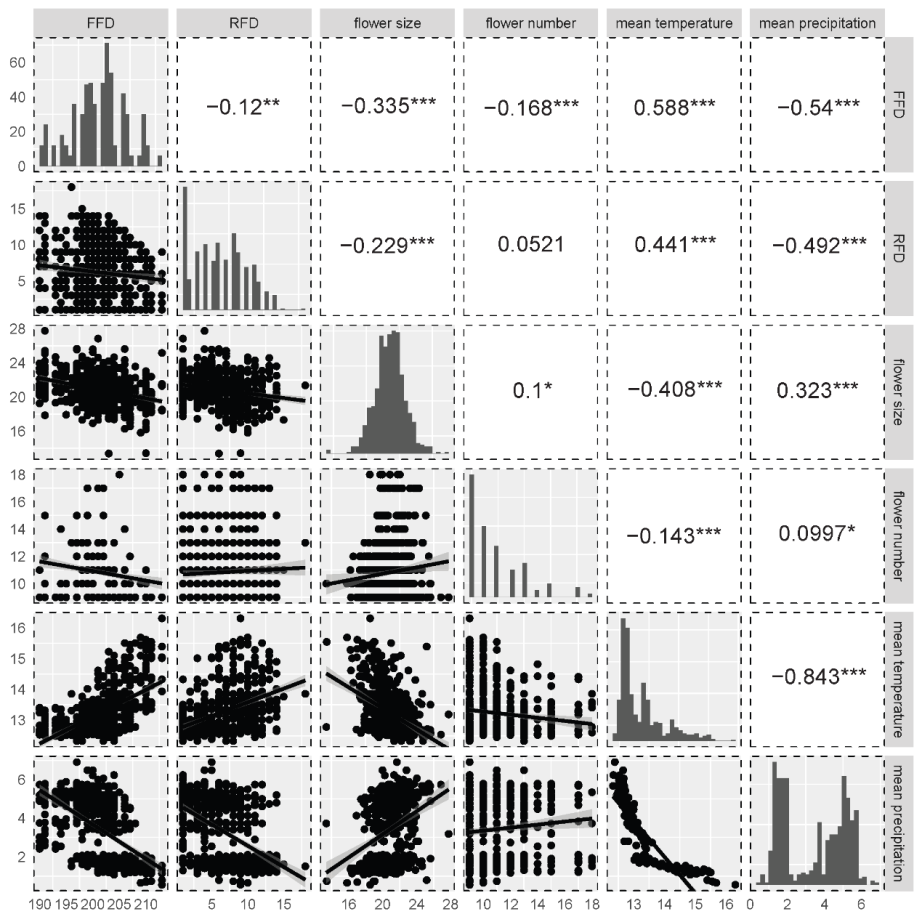


FFD = first flowering date of individuals, RFD = opening day of flowers relative to plant's day of flowering onset; flower size is galea height. Mean temperature and mean precipitation are values experienced by that flower over the days it was open.**P* < 0.05, ***P* < 0.01, ****P* < 0.001
