# Supplementary material for: Phenological variation of flower longevity and duration of sex phases in a protandrous alpine plant: potential causes and fitness significance
Source: BMC Plant Biol. 2020 Apr 3;20:137. doi: 10.1186/s12870-020-02356-w (PMC7118941; doi:10.1186/s12870-020-02356-w)

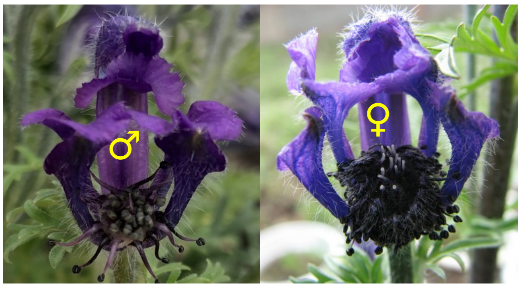

Supplement: Supplementary file 2 [file 12870_2020_2356_MOESM1_ESM.docx]
