# Supplementary material for: Phenological variation of flower longevity and duration of sex phases in a protandrous alpine plant: potential causes and fitness significance
Source: BMC Plant Biol. 2020 Apr 3;20:137. doi: 10.1186/s12870-020-02356-w (PMC7118941; doi:10.1186/s12870-020-02356-w)
Supplement: Supplementary file 3 [file 12870_2020_2356_MOESM2_ESM.docx]

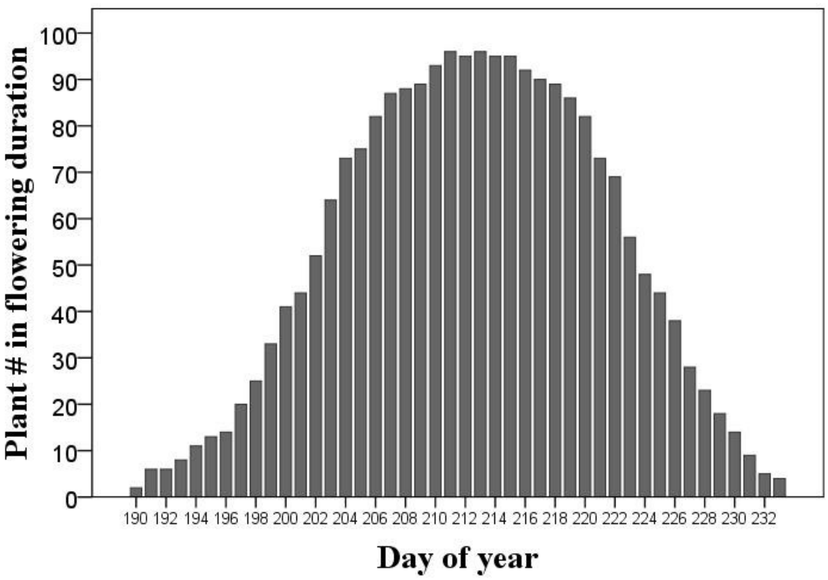

Plant # in flowering duration
100
90
80
70
60
50
40
30
20
10
0
190 192 194 196 198 200 202 204 206 208 210 212 214 216 218 220 222 224 226 228 230 232
Day of year
